# Impact of abusive leader behavior on employee job insecurity: A mediating roles of emotional exhaustion and abusive peer behavior

**DOI:** 10.3389/fpsyg.2022.947258

**Published:** 2022-08-22

**Authors:** Miao Li, Ammar Ahmed, Obed Rashdi Syed, Nadeem Khalid, José E. Muñoz

**Affiliations:** ^1^School of Marxism, Wuhan University of Science and Technology, Wuhan, China; ^2^Department of Management Sciences, MNS-University of Engineering and Technology, Multan, Pakistan; ^3^Sukkur IBA University, Sukkur, Sindh, Pakistan; ^4^Lord Ashcroft International Business School, Anglia Ruskin University, Cambridge, United Kingdom; ^5^Fox School of Business, Temple University, Philadelphia, PA, United States

**Keywords:** abusive leader behavior, abusive peer behavior, emotional exhaustion, job insecurity, serial mediation

## Abstract

Based on the social exchange theory, the present study aimed to investigate the association between abusive leader behavior and job insecurity while considering the serial intervention of abusive peer behavior and emotional exhaustion. Abusive leader behavior triggers abusive peer behaviors, emotional exhaustion, and job insecurity. Results from the data of 323 final responses indicated support for all the hypothesized relationships. Moreover, the findings also reported sequential mediation of abusive peer behavior and emotional exhaustion in the association between abusive leader behavior and job insecurity. The results indicate that mistreatment by an immediate boss can encourage peers to engage in similar unethical behaviors, leading to employees feeling emotionally exhausted, which ultimately results in job insecurity concerns. The study hopes that the findings will help practitioners dedicate more efforts to curtailing abusive behaviors that lead to several unintended consequences at work.

## Introduction

Scholars have been enthusiastic about investigating the behavior of organizational leaders ranging from supervisors to top-level authorities. Organizational leadership deals with a social process of influencing the moods, emotions, actions, and thoughts of the followers/employees/peers. Predominantly, leaders with a positive attitude motivate their followers/peers with a shared vision and activate their inner skills and identities (Johnson et al., 2012). It is commonly believed that leaders are powerful personalities who not only exert influence but also shape the working lives of employees in many aspects (Schyns and Schilling, 2013). It is thought that leaders with great power in organizations also have a great responsibility (Fischer et al., 2021). Specifically, organizational leaders must be careful while exerting their power and refrain from destructive behaviors. The majority of leaders do so and become the main source of increased employee performance. However, some are not and misuse their power by mistreating their employees/followers (Schmid et al., 2019). This study focus on the destructive or dark side of an organizational leader. The most researched form of destructive leadership is also known as “abusive supervision” as defined by Tepper (2000, p. 178) based on a subjective evaluation relying on “subordinates’ perceptions of the extent to which supervisors engage in the sustained display of hostile verbal and non-verbal behaviors, excluding physical contact.” Bies et al. (2016) described the leader as “abusive” if they are continuously involved in mistreating their employees including aggressive verbal and non-verbal behaviors. Scholars have also called them “brutal bosses” (Hornstein, 1996) and “petty tyrants” (Ashforth, 1994). Irrespective of the term used as a leader, their abusive behavior is undesirable. The majority of studies have revealed that abusive supervision is associated with numerous types of negative results related to employees and their organizations (Tepper, 2007; Bies et al., 2016). However, research data are available outlining leaders showcasing behaviors contrary to these, such as hostility toward other employees and subordinates. Over the 22 years, there have been extensive studies to understand the antecedents and outcomes of ill behavior of leaders, such as the use of inappropriate language, outbursts, denouncement, *etcetera* (Tepper et al., 2017). Referring to such behaviors, scholars have used different terms, out of which the most commonly utilized term is abusive leader behavior.

Abusive supervision is one of the unethical practices that lead to organizations’ dysfunction. They put a bad impression on the employees and adversely affect the whole functioning of the organization (Xu et al., 2015; Alzyoud and Odhiabo, 2019). The same goes with the leader and peer abusive behavior. A considerable amount of evidence has been published academically and in public newspapers on supervisor/leader abusive behavior and its increasing occurrence in the workplace (Whitman et al., 2014). This abusive behavior includes public criticism, humiliation, bad temper, sexual harassment, abuse, threatening, injustice, misuse of power, and assigning inappropriate blame (Neves, 2014; Xu et al., 2015). Due to abusive supervisor/leader behavior, organizations face vast hidden costs in decreased organizational citizenship behavior (OCB) and increased counterproductive work behavior (CWB). The broad use of the term abusive supervision is sometimes equated with workplace aggression which has harmful psychological influences on the abused workers (Whitman et al., 2014).

Moreover, in the past decades, researchers have highlighted the issue of supervisor/leader abusive behavior and its consequences (Tepper, 2000), but the fact is that organizations try to cover their leader/supervisor’s unethical practices aimed to avoid a bad reputation in the market. These unethical behavior practices are prevailing in almost all cultures and countries (developed to developing) because most supervisors/bosses/leaders always desire to show the power of their position. It is evident from the organizational behavior studies that most supervisors/leaders use abusive behavior with some targeted employees in their organization because of numerous interpersonal reasons. Some supervisors behave abusively with all organization members because of their leadership style (Neves, 2014).

Importantly, there are gaps in research pertaining to the nexus of abusive leadership and its relationship with triggering abusive peer behavior and emotional exhaustion while affecting job insecurity. Typically, less is known about the internal prospects (mediating) that lead to abusive leader behavior, resulting in unintended consequences at work (e.g., Wang et al., 2019). Moreover, since individuals serving as leaders are often viewed as the guiding light (Boekhorst, 2015), thus there are likely chances that subordinates may imitate the ill behavior and styles, which appears to be requiring further empirical attention. In addition, leader-subordinate relationships often serve as a foundation for an emotional bond (Hon, 2013), yet to what length employees in the high interactive service businesses like the hospitality sector would respond when facing an abusive leader’s behavior remains empirically unattended. Equally, there are questions about the inner mechanism that engenders job insecurity.

Henceforth, the current study objects to making novel contributions to the extant literature on abusive leader behavior and job insecurity. First, though the association between abusive leader behavior and employee outcomes is well attended, the current research investigates its connection with stimulating abusive peer behavior and emotional exhaustion, to which limited empirical attention has been paid. Second, the current study strives to explore the precise inner mechanism that leads to employees developing job insecurity concerns. Third, how these variables interact in the hospitality sector is another interesting occupational gap the current research intends to attend. Fourth, by exploring the theoretical underpinning of the social exchange paradigm (SET), the current study strives further to understand abusive supervision and its negative impact on employees. Fifth, the current study aspires to offer robust implications for practitioners in the hospitality sector, in particular, to understand, recognize and manage the issue of abuse at work.

Given this, the current study proposes to address the gaps mentioned above by investigating three research questions:

RQ1: How does abusive leader behavior sparks abusive peer behavior, emotional exhaustion, and job insecurity?

RQ2: Is there any interceding role of abusive peer behavior in the association between abusive leader behavior and job insecurity?

RQ3: Is there any serially intervening mechanism of abusive leader behavior and emotional exhaustion in the association between abusive leader behavior and job insecurity?

The study used a multi-pronged strategy to respond to these research questions by understanding the various aspects of abusive leader behavior and its consequences by reviewing the existing literature. Then, the study conducted a cross-sectional survey bringing 323 clean responses to investigate the direct associations between abusive leader behavior, abusive peer behavior, emotional exhaustion, and job insecurity. Then, the research investigated the mediation of abusive peer behavior in the nexus between abusive leader behavior and job insecurity. Finally, the study investigated the sequential mediation of abusive peer behavior and emotional exhaustion in the relationship between abusive leader behavior and job insecurity. The remainder of the article is presented as follows: theoretical background and hypotheses development in section 2, followed by data and methods in section 3. Accordingly, section 4 caters to data analysis and interpretation, followed by implications, limitations, and avenues for future research in section 5.

## Theoretical background and hypotheses development

Social exchange theory (SET; Blau, 1968) provides a valuable theoretical understanding to the leader and member relationship and its outcomes. The theory asserts that the relationship between the leaders and subordinates at work is reciprocal and thus, the outcomes of their association rely on the quality of their relationship (Peng et al., 2014; Ahmed et al., 2018). Based on this argument, it is indicated that an employee may experience and showcase poor/unintended behaviors due to negative treatment at work (Guo et al., 2020). Thus, considering abusive leaders providing less trust, respect, support, and mistreatment to employees may trigger others to do the same and develop elements of exhaustion and insecurity. Extant research has also confirmed the suitability of SET in understanding abusive behaviors at work and their negative effects (Zhang and Liu, 2018; Tan et al., 2021; Koay et al., 2022). Moreover, social exchange theory has also served as a notable perspective to investigate factors such as job insecurity (Piccoli et al., 2017) and emotional exhaustion (Cropanzano et al., 2003).

### Abusive leader behavior and abusive peer behavior

In recent times, the abusive behavior of the leader is considered as a destructive leadership style (Guan and Hsu, 2020). The existing organizational behavior literature on abusive supervisors’ behavior is extensive and focuses particularly on the consequences, including the followers’ negative work-related attitudes, co-worker aggression, resistance to work, and deviant work behavior (Tepper et al., 2009; Guan and Hsu, 2020). For instance, various latest studies revealed that supervisor/leader abusive behavior is negatively associated with organizational commitment (Yu et al., 2016; Guan and Hsu, 2020), employee performance (Gan et al., 2020; Shin and Hur, 2020), employee innovative behavior (Wang et al., 2019), employee well-being (Naz, 2020), and positively associated with employee emotional exhaustion (Xu et al., 2015), employee turnover intentions (Liu et al., 2019; Richard et al., 2020), job insecurity (Wang et al., 2019), counterproductive behavior with supervisor (CWB-S; Samreen et al., 2019), counterproductive behavior with non-abused peers (CWB-NP; Samreen et al., 2019), followers interpersonal aggression (Richard et al., 2020), co-worker knowledge hiding behavior (Feng and Wang, 2019). Likewise, it is also revealed that abusive supervision leads to deviant behavior of employees, including violating organizational norms, affecting organizational culture, threatening organization leadership ultimately affecting the smooth functioning of the organization (Kluemper et al., 2019; Park et al., 2019; Guan and Hsu, 2020).

Besides, from the social exchange theory (SET) lens, SET asserts the norm of the exchange of action (Gouldner, 1960). Numerous management and leadership scholars have studied the SET extensively through an employee’s underlying perspective and interactions with their co-worker, supervisor/leader, employer, customers, and supplier, and each relationship has its own situation/background and consequences (Samreen et al., 2019). However, an individual targeted reaction depends on the other side’s action; he/she intends to reciprocate. While leadership studies underpinned by the SET have revealed abusive leader behavior as the most frequent mistreatment practice as perceived by the subordinates, resulting in the change of the discretionary behavior of the subordinates like OCB (Martinko et al., 2013).

Previous studies have established that the consequences of abused supervision are in different forms. The study of Samreen et al. (2019) revealed that the abused employees behave citizenship behavior with the other abused co-workers while behaving counterproductively with the abusive supervisors and non-abused co-workers. This is human psychology and the fact that employees tend to be more favorable toward colleagues who face similar mistreatments rather than toward co-workers who have a good relationship with the supervisors. It is also found that the employees split into groups who face abusive behavior and change their direction from work to other unethical practices to take revenge from the bad bosses (Dalal et al., 2009). Richard et al. (2020) believe that abusive supervision encourages interpersonal aggression among the employees and turnover intention. Apart from the other important consequences like intention to quit, avoiding knowledge sharing, negative job performance, job dissatisfaction, etc., Liu et al. (2019) provided evidence that the supervisor/leader’s abusive behavior contributes to the subordinates/followers’ unethical behavior. It encourages the subordinates to behave unethically in response to supervisor incivility, supporting the norm of SET. Similarly, Wang et al. (2017a) found that employees’ unethical behavior due to the supervisor’s abusive behavior also significantly affects the behavior of other individuals in the organization. This diffusion of the subordinates’ unethical behavior ultimately disrupts the organization’s culture.

In this hypothesis, we assume that when the behavior of a supervisor/leader is abusive, it impacts the behavior of the subordinates in the sense that the individual employee starts behaving in an abusive manner with other co-workers/peers at the workplace. So peers-abusive behavior is a product of the abusive behavior of the supervisor.

Henceforth, it is essential to test social exchange relationships across different sources in organizations. For instance, interpersonal relationships (leader abusive behavior and employee abusive behavior) and targets like (abusive peer behavior) because they are subject to social exchange source-target misalignment (i.e., inconsistent sources and targets of exchange; Mackey et al., 2018).

This hypothesis is drawn on the assumption that organizations, where leaders are supportive produce a supportive work environment and influence co-workers’ behavior to be positive. Thus, we assert that positive leadership helps subordinates develop and display positive behaviors with other subordinates/peers.

Therefore, this hypothesis’s main assertion is that an employee’s behavior becomes abusive with another employee just because his/her supervisor’s behavior was abusive. This hypothesis seeks support from social exchange theory.

*H1*: Abusive leader behavior will be positively related to abusive peer behavior.

### Abusive leader behavior and emotional exhaustion

The impact of abusive leader behavior has multiple consequences for employees. Studies have empirically indicated that any aggression from immediate supervisors and managers drains employee energies and depletes their psychological resourcefulness, thus resulting in work withdrawal (Chi and Liang, 2013). Accordingly, the abusive leader also affects employees’ engagement, which ultimately affects their proactive behavior at work (Stradovnik and Stare, 2018; Wang et al., 2020). Abusive supervision is also viewed as toxic for organizational culture (Tiwari and Jha, 2021), thus having the potential to disturb the entire workflow in a business setup. All this makes it evident how deleterious abusive supervision could be for an organization and its employees. Equally, abusive behavior from leaders or supervisors is empirically established to have a strong association with employees’ emotional exhaustion.

For example, the study by Lim et al. (2020) reported a significant influence of abusive supervision on emotional exhaustion among public sector employees in Malaysia. Similarly, another recent study found abusive supervision as a major workplace stressor causing emotional exhaustion among employees in China (Akram et al., 2019). Accordingly, numerous other studies have also reported similar in the past (Wu, 2008; Peltokorpi, 2019). Taken together, the evidence mentioned above suggests that abusive supervision can trigger fatigue and thus result in emotional exhaustion.

Under this hypothesis, it is asserted that when the behavior of a supervisor is perceived to be abusive. It weakens employees’ emotional stability and psychological state. Employees do not expect support from the supervisor which later on is assumed in a sense that employees start believing that the whole organization does not support him/her. The expected support could be from the professional perspective (i.e., with regards to the task) or personal perspective (i.e., providing social support). Thus, abusive supervision leads employees’ to develop negative perceptions about the organization as a whole in terms of emotional and moral support. Thus, we argue that the consequences of abusive supervision/leadership lead not only toward destructive employee behaviors rather it leads toward employee withdrawal, emotional exhaustion, and the formation of employees’ negative perceptions of the work environment as a whole.

*H2*: Abusive leader behavior will be positively related to emotional exhaustion.

### Abusive leader behavior and job insecurity

As discussed above, the abusive behavior of a leader/supervisor has various harmful consequences. It puts stress on the subordinates, leading to psychological distress and adversely affecting the workplace environment (Wu and Hu, 2013). Over time, when subordinates continuously suffer such hostile behavior, it will make them emotionally exhausted and experience negative emotions relating to burnout, hopelessness, and job insecurity (Wu and Hu, 2013). Job insecurity is one of the individual stressors and refers to the anxiety and fear of an individual losing the existing job (Sverke and Hellgren, 2002). Compared to the actual job loss, job insecurity is the perception of the employee that his/her job is at risk. It is fact that psychologically disturbed employees will lose their ability to cope with workplace challenges and threats. Eventually, it increases the employee’s sense of job insecurity.

Working in this critical environment, employees start experiencing negative emotions and produce counterproductive behavior. Those employees who are under psychological pressure will get involved in such harmful practices that affect their reputation at the workplace and work-life balance. The pertinent literature related to the abusive behavior of leadership acclaimed that the victims of supervisor abusive behavior also get pressured to quit the job or resign from the job (Haar et al., 2016; Mathieu and Babiak, 2016; Arif et al., 2017; Xu et al., 2018; Shin and Hur, 2020). Those abusive behaviors from leadership deteriorate the skills of the employees, and he/she could not able to stand along with their co-workers, which at last makes him/her feeling job insecure (Wang et al., 2019). It is also observed that employees who face supervisor abusive behavior regularly get feelings of helplessness and isolation from their co-workers and organization.

Under this hypothesis, we assume that when the behavior of a supervisor is abusive it does not only affect employees’ negative perception formation but influences attitudes of the employees where employees could possibly develop job insecurity because employees start seeing abusive behavior as a threat to not only their well-being but also to their jobs. Thus, with abusive supervision or leadership behavior, job insecurity increases.

*H3*: Abusive leader behavior will be positively related to job insecurity.

### Abusive peer behavior and emotional exhaustion

Hostile treatment at the workplace does not necessarily come from the leaders only. Scholars have argued that all the unintended consequences at work also have a substantial contribution from the behavior of peers. Therein, how on one side, positive prospects from peers at work bolsters employee behaviors and outcomes (Ahmed et al., 2016; Shin et al., 2020), the same way abhorrent behaviors negatively affect (Tews et al., 2019). This is why the behavior of peers and the working environment with colleagues and co-workers are instrumental to ensuring employees can serve organizational goals. When peers engage in abusive behaviors such as maltreatment or ridiculing other employees, this may lead to losing connectivity with the work and organization. Thus, such a situation will make it hard for an employee to express psychological resourcefulness at work.

Based on this, we argue that, in the face of abusive peer behavior, employees may not be able to express connectivity with the job or feel comfortable about their work roles. Any unwarranted behavior from co-workers, such as insults, can affect employee behaviors and outcomes, i.e., emotionally exhausted (Askew and Buckner, 2017). A meta-analysis has reported it as one of the deviant work behaviors and pinpointed its potential dangers for others in the business (Pletzer et al., 2020). Hostility in the workplace faced by peers is equally harmful to other employees and the organization (Einarsen and Mikkelsen, 2002). Accordingly, depending on the length of time an individual has been experiencing abusive behavior from peers, it can significantly damage individual identity and emotions.

Therefore, based on the pertinent literature, the present study hypotheses that abusive peer behavior not only physically affects the co-workers but also emotionally exhausts them which leads to poor performance and other psychological issues. This leads to the development of the following hypothesis:

*H4*: Abusive peer behavior will be positively related to emotional exhaustion.

### Abusive peer behavior and job insecurity

Investigating job insecurity is a continuing concern within the field of organizational behavior and holds enormous importance (Shoss, 2017). While in relationship to the abusive peer behavior and/or workplace bullying literature, the job insecurity variable has been ignored (Sarwar et al., 2020). Job insecurity is conceptualized as an employee’s fear of job loss, a threat of losing necessary job features, and the need to maintain his/her job (Greenhalgh and Rosenblatt, 1984; Jiang and Probst, 2016). According to Vander Elst et al. (2016), job insecurity is the employee’s perceived threat of losing the current job in the coming future. Even though those employees are treated with respect and dignity at the workplace feel job security (Lee et al., 2018). In the pertinent literature relating to job insecurity, there are numerous reasons for the perceived job insecurity, including environmental fears (Klandermans et al., 2010). For instance, job insecurity tends to increase with the increase in the unemployment rate, changes in the business environment or technological changes, the decline in occupational skills, and shrinking demand for labor (Jiang et al., 2013; Lübke and Erlinghagen, 2014). Whereas, at the organizational level, a decrease in organizational performance, changes in management, and formal and informal announcements regarding the changes in the labor policies serve as a warning sign that one’s job might be at risk, consequently increasing job insecurity (Debus et al., 2014; Ellonen and Nätti, 2015).

On the other hand, individual factors can also source perceived job insecurity. According to Debus et al. (2014), the person-related variables and environmental factors directly contribute to perceived job insecurity. When an individual does not find himself/herself person-environment fit, he/she gets the negative vibes that directly threaten him/her. This threat leads to emotional exhaustion, absenteeism, low self-esteem, and job insecurity. Previous research has devoted much attention to the consequences of job insecurity, while little attention has been paid to the predictors/antecedents of job insecurity (Shoss, 2017; Li et al., 2020). The meta-analysis concerning the predictors of job insecurity emphasized the need to examine the psychosocial stressors that trigger the feelings of job insecurity (Keim et al., 2014).

Whereas, Shoss (2017), in his integrative review, recommended future scholars investigate the additional workplace stressors that can enhance the perception of job insecurity. Workplace anxiety is one workplace stressor that triggers and disturbs the organizational culture (Li and Ju, 2018). When there is workplace anxiety and co-workers did not provide support to their peers and mistreat their co-workers due to weak organizational culture, it ultimately leads to the fear of losing the current job. Jiang and Lavaysse (2018) found that positive and negative peer support directly affects cognitive and affective job insecurity. Positive peer support is an ethical behavior from one employee to another employee, which shows a strong organizational culture is the main contributor to the employee’s performance. In comparison, negative peer support shows unethical/abusive peer behavior that adversely affects the co-worker’s emotions and cognitive abilities. Tomas et al. (2019) also support that co-worker support is negatively related to job insecurity.

In addition, the study of Li et al. (2020) examined the impact of workplace mistreatment on job insecurity. They examined the role of abusive supervisor behavior, workplace bullying, and workplace incivility as main predictors of job insecurity. Nonetheless, little attention has been paid to examining workplace mistreatment as a predictor of job insecurity. Hence, based on the theoretical and empirical shreds of evidence, it is assumed that abusive peer behavior develops the perception of job insecurity. The current study focuses on abusive peer behavior and its relationship with job insecurity to contribute to this perspective. Furthermore, from a social exchange viewpoint, the relationship between employer-employee and employee-to-employee is based on exchange relationships. When employees are mistreated either by the leader/supervisor and/or by the peer, it reciprocates in negative results (Shoss, 2017).

Therefore, it is concluded that attitudes toward anything are almost always based upon seeing some consistent patterns in behavior. When an employee finds that co-workers are abusive rather than cooperative, it results in a lack of cooperation, trust, and teamwork. Thus, the absence of these factors fosters individuals to believe that their work is not secure at all. Therefore, we argue as under:

*H5*: Abusive peer behavior will be positively related to job insecurity.

### Emotional exhaustion and job insecurity

Employees always believe that organization will support them in case any turbulence or imbalance situation occurs. Perceived organizational support is employees’ perception regarding the support from the organization in terms of actions taken against employees (Eisenberger et al., 2001). Perceived environmental support provides hope to the employees that the organization will take care of them and reciprocate their efforts in terms of rewards and perks, and save them from any insecurity. Leaders being representative of the organization can enhance the employees’ perception and emotions by meeting the employees’ expectations (Dirican and Erdil, 2020). In contrast, supervisor abusive behavior or ignorant behavior toward employees reduces the perception of organizational support and gives rise to emotional exhaustion that leading to job insecurity.

Moreover, perceived environmental support contains a meaningful employee-organization relationship and deals with a variety of implications, including the peer’s supportive behavior, employee well-being, and positive behavior toward the organization (Eisenberger et al., 2019). Previous research has provided evidence on the relationship between abusive supervision and perceived organizational support (Xu et al., 2018; Dirican and Erdil, 2020). At the workplace, employees face different challenges and imbalance situations. Being the main contributor in the organizational performance/development, he/she always expects support from the organizational leadership. When an organization fails to provide a safe environment or support to the employees when they need it, they emotionally get exhausted and the perceived job insecurity develops. For instance, Tomas et al. (2019) analyzed the psychological climate dimensions as antecedents of job insecurity through occupational self-efficacy. The psychological climate dimensions include job challenge, leader support, role harmony, and co-worker cooperation. The authors believed that these four factors are negatively related to job insecurity.

Furthermore, Ali et al. (2020) discussed the work-related outcomes due to job insecurity. It involves negative behaviors like disengagement, burnout, dissatisfaction, lower organizational commitment, a decline in employee innovation, and employee performance. In addition to that, an organization bears a high cost in terms of lowered productivity. Although, organizations gain numerous advantages from their employees in exchange for their organizational support and ethical leadership. Emotional exhaustion in employees develops job insecurity; this assertion is very much supported by social exchange theory. But, it goes the opposite; when organizational support is provided through ethical supervision and supportive behavior is performed, employees find themselves emotionally strong and secure.

Therefore, it is necessary to empirically examine the relationship between emotional exhaustion and job insecurity.

*H6*: Emotional exhaustion will be positively related to job insecurity.

### Mediation of abusive peer behavior

As discussed earlier, abusive supervisor/leader behavior is the sustained hostile verbal and non-verbal behavior perceived by the subordinates/co-workers. It is evident from the previous literature that supervisor/leader abusive behavior negatively affects the work environment, subordinates’ personal traits, and co-workers’ performance (Yang et al., 2019). When a leader/supervisor shows abusive behavior, every employee responds to it differently according to his/her perception of the abuse. It is also supported by the previous literature that sustained abusive supervisor behavior develops perceived job insecurity among subordinates (Haar et al., 2016; Mathieu and Babiak, 2016; Arif et al., 2017; Xu et al., 2018; Shin and Hur, 2020). Nonetheless, one more factor contributing to the rise of perceived job insecurity is abusive peer behavior. Keim et al. (2014) and Shoss (2017) argued that workplace stressors and psychosocial stressors trigger developing perceived job insecurity. When employees continuously face the supervisor/leader’s abusive behavior, it affects the work environment, leading to the abusive behavior of co-workers. Although abusive supervisor behavior has many consequences, including job security issues, it also develops co-workers’ abusive behavior. A Supervisor/leader is a role model for any organization as employees directly learn from him/her personality. When they experience negative behavior from the leader, they believe that this is the right way to get things done.

Similarly, the abusive peer behavior will more adversely affect the co-worker’s motivation; they do not find any support from their co-workers. Hence, this lack of support from the supervisor and peers develops the feeling of job insecurity. According to Debus et al. (2014), interpersonal-related factors have a double impact on perceived job insecurity than environmental-related factors. Some employees did not find a person-environment fit in the organization, thus leading to job security issues. Since abusive supervision alone leaves an impact on an employee’s belief that a given workplace is not secure. This assumption of an employee is further strengthened if he/she finds a similar pattern in the behaviors of the co-workers. Co-workers are significant in a given environment because they could hear employee problems, guide on what to do, share workload-if and when needed- and give you moral support in difficult times.

On the contrary, when you as an employee find that your supervisor’s behavior is abusive and the co-workers have been badly treated, it helps you develop an opinion that perhaps this whole system is against you and perhaps your appointment in the workplace was against their wish and will. Thus, behaviors like these become the basis for forming negative attitudes about others, and these could potentially cause job insecurity. Furthermore, abusive peer behavior as a mediator has been rarely examined, while it works as a primary trigger that activates the perceived job insecurity (Shoss, 2017).

Considering the whole scenario, the present study fills this gap and contributes to the pertinent literature on job insecurity by considering abusive peer behavior as a mediator between abusive supervision and job insecurity relationship. Based on the above discussion, the following hypothesis has been proposed.

*H7*: The relationship between abusive supervision and job insecurity is mediated by abusive peer behavior.

### Sequential mediation of abusive peer behavior and emotional exhaustion

Literature on job insecurity highlighted person-related variables and environmental-related factors as predictors/antecedents of job insecurity (Debus et al., 2014; Shoss, 2017). Even though numerous studies have been conducted on the consequences of job insecurity (Sarwar et al., 2020), the studies examining the antecedents/factors that develop job insecurity are limited (Keim et al., 2014; Shoss, 2017). Mistreatment of employees, including workplace incivility, abusive supervisor/leader behavior, and workplace bullying (personal bullying, physical bullying, and work-related bullying), emotional instability shows a lack of work centrality in the organization (Li et al., 2020). Nonetheless, abusive supervision and abusive peer behavior develop the fear of job insecurity. But under that circumstances, employees look for support from the environment/organization. When he/she found a lack of perceived environmental support then he/she experienced different types of fears and issues including job insecurity. Organizational politics is also one environmental factor that develops the fear of job insecurity (Hu, 2010).

**Figure 1:**
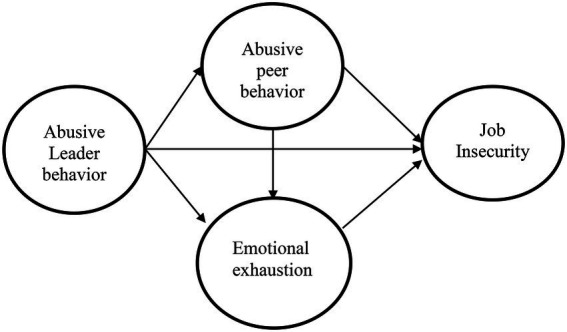
Research framework.

In this case, when an employee perceives that his/her boss has an abusive style and the same is found in their peers; it creates emotional exhaustion and employees feel a lack of support from the environment, which enhances job insecurity. In the present study case, a lack of environmental support and abusive supervisor behavior develops emotional instability and the employee feels exhausted. In other words, this feeling of an employee that an organization’s environment provides no support is developed because of the abusive leadership behavior and abusive peer behavior. We assert that abusive leadership behavior alone might not be that much harmful compared to when it’s combined with abusive peer behavior. In a situation like that, an employee feels lonely, could not find empathy, and sees no way to improve his/her work performance. Keeping the point of conservation of resources theory, the continued practices of mistreatment/abusive supervision at the workplace reasoned the decline in the psychological resources of employees (i.e., emotional strength and job insecurity; Akram et al., 2019; Wang et al., 2019). Thus, situations like that affect employees emotionally, and employees feel insecure in the workplace. Although extensive research has been carried out on job insecurity, any rare study exists that examined the role of abusive peer behavior and emotional exhaustion as a sequential mediator. Moreover, the social exchange perspective also underpinned the proposed mechanism. Hence, it is necessary to investigate the role of emotional exhaustion along with the person-related factor (peer abusive behavior) as a mediator between abusive supervision and job insecurity. Based on that, the following hypothesis is formulated and the conceptual framework is depicted in Figure 1.

*H8*: The relationship between abusive supervision and job insecurity is sequentially mediated by abusive peer behavior and emotional exhaustion.

## Methodology

### Data collection and sample

The nature of this study was cross-sectional and data were collected from the employees in the hotel sector of Pakistan. However, keeping in view the considerations to obtain a minimum required response, we distributed 500 questionnaires among respondents. Data collection was done through using questionnaire with self-reporting measures. HR units were requested to help assist in this regard to approach desires respondents. Some of these questionnaires came incomplete thus those were discarded, other respondents did not fill the questionnaire at all. Therefore, as a result of our data collection efforts, we successfully obtained 323 cleaned responses that were used for analysis. Out of the 323 respondents, 189 were male and 134 were female. In connection to education, 94 reported to have high school degree or advanced diploma, 211 with bachelor’s degree and 18 having a master degree. Furthermore, the original draft of the questionnaire was in English, whereas, no translation procedure was required because English is the official teaching language in higher education sectors of Pakistan.

### Measures

We measured all the items on a 5-point Likert Scale, and the measures were adopted from the published sources. Abusive leader behavior and abusive peer behavior were measured using fifteen item scale adopted from the study (Tepper, 2000). A sample item “Does not allow me to interact with my co-workers.” However, the abusive peer behavior questionnaire was slightly changed. For example, we replaced the supervisor term with the peer, and the finalized questionnaire was pre-tested with the actual respondents. Emotional exhaustion was measured using a nine-item scale from Maslach and Jackson (1981). This instrument of emotional exhaustion has been extensively used in the literature to measure emotional exhaustion. Lastly, job insecurity was measured using a 3-item scale from Hellgren and Sverke (2003).

Peer abusive behavior was assessed with the four-item scale about direct victimization by (Aquino et al., 2004). The workplace victimization scale has two dimensions, including direct and indirect victimization. Because direct victimization is more salient than indirect victimization and it is more consistent with our research question, we used only the direct victimization scale in our research. Participants were asked to indicate their agreement regarding whether they had experienced the listed actions from their co-workers (1 = “strongly disagree,” 6 = “strongly agree”). Sample items included “Your coworkers made an obscene comment or gesture in front of you” and “Your coworkers swore at you.”

### Common method bias

The study deployed Harman’s single-factor analysis to assess common method bias. This approach proposed by Harman (1976) affirms whether or not the variations in the data are accounted for by only one variable. Therein, if a single variable accounts for more than 50 percent of the variance in the data, then it confirms the issue of common method bias (Podsakoff et al., 2003). Results from the rotated factor matrix did not indicate any factor accounting for more than 50%. Henceforth, there was no potential problem of common method bias in the study.

## Results

### Data analysis technique

PLS-SEM has gained popularity recently (Refer Hair et al., 2019). This tool is beneficial when the purpose of the study is to predict relationships (McDonald, 1996, p. 240; Ringle et al., 2015; Hair et al., 2019; Ahmed et al., 2021). Thus the current study was analyzed using SmartPLS software using a two-step approach (Henseler et al., 2009). Following Henseler et al. (2009), we examined individual item reliability through outer loadings and retained items with 0.50 loadings or above (Duarte et al., 2010; Hair et al., 2014). We then analyzed internal consistency reliability by looking into composite reliability (CR) and Cronbach’s alpha and found values for each of the latent variables of the model above the minimum threshold of 0.70 (Bagozzi and Yi, 1988; Hair et al., 2011). Next, we found that the average variance extracted (AVE) for each construct was greater than 0.50 (Chin, 1998; Hair et al., 2016). These results are provided in Table 1. Lastly, we examined discriminant validity using Heterotrait-monotrait-HTMT approach. Our results indicated that all the HTMT values were under 0.90 (Kline, 2011). Thus discriminant validity was not a problem in this study (Table 2).

**Table 1:** Measurement model.

Construct	Item	Loadings	Alpha	CR	AVE
Abusive peer behavior	AB10	0.862	0.952	0.956	0.647
	AB11	0.718			
	AB12	0.845			
	AB13	0.865			
	AB14	0.839			
	AB2	0.828			
	AB3	0.702			
	AB4	0.840			
	AB5	0.718			
	AB6	0.854			
	AB8	0.847			
	AB9	0.707			
Abusive leader behavior	ALB1	0.813	0.917	0.93	0.551
	ALB11	0.644			
	ALB13	0.781			
	ALB14	0.615			
	ALB15	0.622			
	ALB2	0.812			
	ALB3	0.716			
	ALB4	0.652			
	ALB6	0.791			
	ALB8	0.823			
	ALB9	0.838			
Job insecurity	JI1	0.845	0.778	0.871	0.692
	JI2	0.840			
	JI3	0.811			
Emotional exhaustion	EOS2	0.772	0.925	0.943	0.735
	EOS3	0.909			
	EOS4	0.925			
	EOS5	0.693			
	EOS6	0.917			
	EOS7	0.900			

**Table 2:** HTMT.

Constructs	Abusive leader behavior	Abusive peer behavior	Emotional exhaustion	Job insecurity
Abusive peer behavior	0.560			
Emotional exhaustion	0.740	0.533		
Job insecurity	0.788	0.815	0.890	–

### Structural model assessment

The explanatory power of the model was evaluated using *R*^2^ values. Accordingly, we found *r*-squared values for abusive peer behavior, emotional exhaustion, and job insecurity as 0.34, 0.51, and 0.77, respectively (Table 3). Next, we examined *f*-square values to find out the model’s explanatory power (Hair et al., 2019). We found that abusive leader behavior’s effect on abusive peer behavior and emotional exhaustion was substantial; however, its effect on job insecurity was weak. Next, abusive peer behavior’s effect on emotional exhaustion was weak, whereas its effect on job insecurity was strong. Lastly, we found that emotional exhaustion’s effect on job insecurity was strong. These findings related to *f*-squared assessment are drawn upon guidelines suggested by Hair et al. (2019).

**Table 3:** *R*^2^ and *Q*^2^ values.

Constructs	SSO	SSE	*Q*^2^ (=1-SSE/SSO)	*R* ^2^
Abusive peer behavior	3,876	3,141.299	0.190	0.345
Emotional exhaustion	1,938	1,234.616	0.363	0.516
Job insecurity	969	460.751	0.525	0.772

In order to report predictive power, we examined a cross-validated measure of redundancy (*Q*^2^). This assessment is recommended in the literature (refer to Chin, 2010; Ringle et al., 2012; Hair et al., 2013). Particularly, Henseler et al. (2009) recommended that if the *q*-squared value for the dependent variable(s) is greater than zero, the model demonstrates predictive relevance. Based on these guidelines, we found that *q*-squared values for all of our dependent variables were greater than zero.

The structure model evaluation was carried out using bootstrapping approach with 5,000 subsamples (Hair et al., 2016), and results are shown in Table 4. Our first hypothesis was related to the direct relationship between abusive leader behavior and abusive peer behavior, for which we found empirical support (*β* = 0.587, *t* = 18.15, *p* < 0.01). In H2, we claimed a positive relationship between abusive leader behavior and emotional exhaustion and found support (*β* = 0.584, *t* = 13.129, *p* < 0.01). In H3, we hypothesized a positive relationship between abusive leader behavior and job insecurity (*β* = 0.131, *t* = 3.655, *p* < 0.01). In H4, we stated a positive relationship between abusive peer behavior and emotional exhaustion (*β* = 0.198, *t* = 4.210, *p* < 0.01) and found support. In hypothesis 5, we stated a positive relationship between abusive peer behavior and job insecurity (*β* = 0.451, *t* = 15.797, *p* < 0.01) and found support. Lastly, in H6, we stated that emotional exhaustion would be positively related to job insecurity (*β* = 0.435, *t* = 10.529, *p* < 0.01) and found empirical support.

**Table 4:** Path coefficients direct relationships.

Hypo	Relationships	Beta	*t*-value	BCI LL	BCI UL	*f*	Decision
H1	Abusive leader behavior → Abusive peer behavior	0.587	18.15	0.536	0.642	0.527	Supported
H2	Abusive leader behavior → Emotional exhaustion	0.584	13.129	0.509	0.657	0.462	Supported
H3	Abusive leader behavior → Job insecurity	0.131	3.655	0.072	0.191	0.034	Supported
H4	Abusive peer behavior → Emotional exhaustion	0.198	4.221	0.119	0.274	0.053	Supported
H5	Abusive peer behavior → Job insecurity	0.451	15.797	0.406	0.500	0.554	Supported
H6	Emotional exhaustion → Job insecurity	0.435	10.529	0.361	0.498	0.401	Supported

### Mediation analysis

In the present study, the mediation analysis was carried out using bootstrapping procedure as Preacher and Hayes (2004, 2008) suggested using 5,000 subsamples to determine indirect effects. The findings are presented in Table 5. These approaches have been widely used across industries (Ahmed et al., 2021). We hypothesized, in H7, that the direct relationship between abusive leader behavior and job insecurity was mediated by abusive peer behavior (*β* = 0.265, *t* = 16.068, *p* < 0.01; LL = 0.24, UL = 0.295). Thus, H7 was supported. Similarly, we hypothesized a serial mediation between abusive leader behavior, abusive peer behavior, emotional exhaustion, and job insecurity and found empirical support (*β* = 0.051, *t* = 3.680, *p* < 0.01; LL = 0.029, UL = 0.074).

**Table 5:** Indirect paths (mediation analysis).

Hypo	Relationships	Beta	*t*-value	BCI LL	BCI UL	Decision
H7	Abusive leader behavior → Abusive peer behavior → Job insecurity	0.265	16.068	0.24	0.295	Supported
H8	Abusive leader behavior → Abusive peer behavior → Emotional exhaustion → Job insecurity	0.051	3.68	0.029	0.074	Supported

## Discussion

The study results demonstrate how abusive behaviors at work could be detrimental to individual behaviors and outcomes. The study’s findings confirmed the significant relationship between abusive leader behavior and abusive peer behavior. The result suggests that abusive leader behavior contributes to unethical and abusive behaviors of the followers/peers. The findings are consistent with the limited empirical evidence on this association (Wang et al., 2017b; Liu et al., 2019). Workplaces with hostile behaviors from leaders can result in interpersonal aggression among the general employees (Richard et al., 2020), thus leading to unintended individual and organizational outcomes. Accordingly, the current study reported a significant impact of abusive leaders on employees’ emotional exhaustion. The findings indicate that employees who experienced abusive treatment from their leaders ended with emotional trauma and disturbance. The results agree with prominent studies (Wu, 2008; Peltokorpi, 2019) indicating the deleterious effects of abusive leader behavior. Since interpersonal relationships with supervisors are of high importance (Ahmed et al., 2016) thus, any unintended or ill behavior from them can easily result in emotional exhaustion (Akram et al., 2019).

Furthermore, the study also reported the toxic effects of abusive peer behavior on employees` sense of security. As a work stressor (Wu and Hu, 2013), it can make employees develop concerns about their job, thus experiencing job insecurity. Although compared to actual job loss, job insecurity is just a perception of an employee that his/her job is at risk (Wang et al., 2019) yet still, it can result in several unintended consequences (Haar et al., 2016; Mathieu and Babiak, 2016; Arif et al., 2017; Xu et al., 2018; Shin and Hur, 2020). In parallel, abusive peer behavior can also contribute to employees’ emotional exhaustion. The findings confirm the negative impact of hostility from leaders and peers at work (Einarsen and Mikkelsen, 2002; Askew and Buckner, 2017) and how it can further employees` emotional exhaustion.

Similarly, the findings also confirmed the effect of abusive peer behavior on the feelings of job insecurity, suggesting that mistreatment from peers can damage employees` confidence in the job and work roles. The findings help understand some of the prior studies (e.g., Li and Ju, 2018) on how it can damage work culture that later develops job insecurity among employees (Jiang and Lavaysse, 2018; Tomas et al., 2019). Lastly, for direct relationships, emotional exhaustion also contributed to job insecurity. The significant statistical results suggest that when employees are psychologically detached and suffering distress at work, it makes them develop concerns about their job. This confirms the empirical conclusions of Tomas et al. (2019) and Ali et al. (2020) about the harmful effects of psychological exhaustion at work on employee behaviors and outcomes, including job insecurity.

Accordingly, the study found abusive peer behavior mediating the association between abusive leader behavior and job insecurity. The findings indicate that leaders’ ill-treatment can trigger employees to misbehave with others, adding to their job insecurity. Viewing abusive peer behavior as a workplace stressor (Shin and Hur, 2020), the findings confirm the empirical assertions of Keim et al. (2014) and Shoss (2017) as to how workplace stressors can result in the development of job insecurity. The findings are also novel as, to the best of our knowledge; it is the first study testing the mediation of abusive peer behavior in the abusive leader behavior and job insecurity association. The statistical results also found that abusive leader behavior and job insecurity were sequentially mediated by abusive peer behavior and emotional exhaustion. The findings indicate a deeper engendering whereby poor leaders’ treatment can result in encouraging peer of behaving the same way, which can develop emotional trauma in employees, thus predicting job insecurity. This serial connection outlines the toxic effects of abusive leader behavior that affect and spoil several elements in a business environment and concludes with developing perceptions of job insecurity among the employees.

## Implications

### Theoretical implications

The study has several important theoretical implications. First, the study has contributed to confirming the assertions of social exchange theory and how these exchange-based relations influence individual behaviors at work. The findings outline that maltreatment by leaders/managers can ignite many unhealthy behaviors at work, resulting in many unintended behaviors and outcomes. Secondly, the current study has broadened our understanding of the consequences of abusive leader behavior and its relationship with job insecurity. In addition, the study has also responded to calls outlining the need for further investigation on the antecedents of job insecurity (e.g., Shoss, 2017). Third, although abusive peer behavior is termed a workplace stressor (Shin and Hur, 2020), no empirical evidence was found to test the mediation of abusive peer behavior in the relationship between abusive leader behavior and job insecurity. Fourth, the study has also contributed to outlining the serial engendering effect of abusive leader behavior and emotional exhaustion in the relationship between abusive leader behavior and job insecurity. Hence, our study contributes to literature by linking abusive leader, peer, motional exhaustion and job insecurity in the hotel sector. Likewise, our research contributes to extant knowledge by showing that emotional exhaustion, a labor issue (Choi et al., 2014), can significantly detach employees from work (Hellgren et al., 1999; Selenko et al., 2017) through developing job insecurity among employees.

### Practical implications

Some important implications for practitioners can be drawn from the study’s findings. First, the study indicates that tackling job insecurity potentially requires organizations to consider working abusive leader behavior. Although abusive behavior from bosses at times is due to some specific reasons (Khan et al., 2018), it still needs to be minimized to control abusive peer behavior. For this, based on some empirical evidence (Lau, 2010; Gonzalez-Morales et al., 2018), we imply the use of training interventions to tackle ethical issues among leaders. Accordingly, the HR department can also play an important role in developing objective policies outlining the serious consequences of such behaviors (Wang et al., 2019).

Second, the findings indicated that ill-treatment from both leaders and peers is also important to be controlled due to its strong contribution to draining employees’ psychological capabilities, resulting in employees feeling strained and exhausted. The study implies that hotels experiencing this may consider working on improving work culture and control power culture. This is based on the empirical evidence suggesting that workplaces with high power distance perception in connection to leaders and supervisors often result in emotional exhaustion (Trzebiatowski and Triana, 2020).

Third, the study implies that organizations to consider investigating employees’ concerns about their job insecurity. Some empirical evidence suggests that developing trust-based relationships is essential to avoiding job insecurity (Richter et al., 2018). Therefore, we suggest hotels examine the extent to which employees trust each other and their leaders.

Fourth, there should be channels for assistance available for employees experiencing abusive behaviors to resolve any psychological or behavioral issues that they might be facing. For example, employee assistance programs or the establishment of counseling units (Arthur, 2000; Kirk and Brown, 2003). Such initiatives can help improve employee morals, tackle job insecurity, and cultivate a positive working culture.

## Limitations and scope for future research

Despite notable contributions to theory and practice, some limitations are highlighted for future scholars to consider. In the views of Bloom and Reenen (2010), management practices are likely to differ across organizations, economies, and business sectors. Based on this assertion, there are likely chances that the nature of abusive leader behavior and its consequences vary across organizations, business sectors, and economies. Therefore, the results of the current study pose a narrow opportunity when it comes to the generalizability of the results since it only focused on the hospitality sector in a single geographical location (metropolitan cities of Pakistan). Scholars in the future may find it interesting to validate the results across other emerged and emerging economies. Moreover, the present study investigated the serial mediation of abusive peer behavior and emotional exhaustion in the abusive leader behavior and job insecurity association. We argue that the present study’s explanatory power may improve by testing other theoretical mediators (i.e., burnout, fatigue, anxiety) and moderators (i.e., family motivation, self-efficacy, and resilience). Accordingly, it is empirically proven that abusive leader behavior has unfavorable effects on employees and the whole work environment; however, what triggers leaders to behave in this way remain unclear. Therefore future scholars may consider investigating the root causes of abusive leader behavior. Equally, how abusive leader behavior can be controlled or eradicated is another area requiring urgent empirical attention to cultivate a healthy work environment.

## Data availability statement

The raw data supporting the conclusions of this article will be made available by the authors, without undue reservation.

## Ethics statement

The studies involving human participants were reviewed and approved by MNS-University of Engineering and Technology, Multan, Pakistan. The patients/participants provided their written informed consent to participate in this study.

## Author contributions

AA and ML proposed the research idea, analyzed the results, and wrote the manuscript. OS, NK, and JM carried out the methodology and extensively edited the manuscript. All authors contributed to the article and approved the submitted version.

## Funding

This paper was supported by Hubei Education Planning General project 2021GB013.

## Conflict of interest

The authors declare that the research was conducted in the absence of any commercial or financial relationships that could be construed as a potential conflict of interest.

## Publisher’s note

All claims expressed in this article are solely those of the authors and do not necessarily represent those of their affiliated organizations, or those of the publisher, the editors and the reviewers. Any product that may be evaluated in this article, or claim that may be made by its manufacturer, is not guaranteed or endorsed by the publisher.
